# Use and impact of digital technology in supporting health providers deliver care in low- and low-middle-income countries: A systematic review protocol

**DOI:** 10.1371/journal.pone.0319190

**Published:** 2025-02-21

**Authors:** Adam T. Craig, Harriet Lawford, Maggie Miller, Liuyi Chen-Cao, Leanna Woods, Siaw-Teng Liaw, Myron A. Godinho

**Affiliations:** 1 Centre for Clinical Research, The University of Queensland, Brisbane, Queensland, Australia; 2 National Centre for Epidemiology and Population Health, Australian National University, Acton, Australian Capital Territory, Australia; 3 Queensland Digital Health Centre, The University of Queensland, Brisbane, Queensland, Australia; 4 School of Population Health, University of New South Wales, Sydney, New South Wales, Australia; 5 Westmead Applied Research Centre, The University of Sydney, Westmead, Australia; Haramaya University, ETHIOPIA

## Abstract

**Background:**

Healthcare providers are critical to the successful, sustainable and impactful implementation of digital health. Despite the growing interest, Digital Health Innovations (DHIs) are often implemented without sufficient evidence, leading to numerous short-lived projects and a limited understanding of their impact on health systems and outcomes. In 2023, the World Health Organization (WHO) introduced a new *Classification of Digital Health Interventions*, identifying four core DHI user groups. This review aims to synthesise evidence on the impact of DHIs designed for one of these user groups—healthcare providers—on delivering health services in low- and lower-middle-income countries (LLMICs).

**Methods:**

We will conduct an umbrella review, analysing systematic reviews on DHIs for healthcare providers. Data will be extracted using deductive coding before being thematically analysed according to the 11 DHIs for service providers outlined in the WHO’s 2023 Classification.

**Discussion:**

This umbrella review will explore, synthesise, and evaluate the quality of evidence on how healthcare providers in LLMICs utilise DHIs to address service delivery challenges and their effectiveness. To our knowledge, this is the first comprehensive synthesis of evidence focused on DHIs designed for use by healthcare providers in LLMICs. It is also the first review to align with WHO’s taxonomy for DHIs, as outlined in the WHO *Classification of Digital Interventions, Services and Applications in Health.*

**Systematic review registration:**

The protocol is being registered in PROSPERO (ID: CRD42024586285).

## Introduction

The digital revolution has ushered in a new era of possibilities for healthcare systems worldwide, from electronic health records and telemedicine to artificial intelligence (AI) in medical diagnosis and remote patient monitoring. In the evolving healthcare ecosystem, healthcare providers have emerged at the forefront of this transformation, leveraging digital technologies to navigate and address the multifaceted challenges faced in delivering care when and where it is required.

The adoption of digital technology within healthcare has been fuelled by several factors: cost-effectiveness, demand for data-driven decision-making, technology advancements, and new analytical methods (such as machine learning). These factors have opened opportunities to gather, process, exchange, and analyse medical and health service data securely and in near real-time. This has aided the delivery of data-driven clinical care and service management in developed and developing countries, with governments, donors, and multilateral institutions recognising the opportunity digital technologies offer. In May 2018, governments unanimously adopted a World Health Assembly resolution that called for the World Health Organization (WHO) to develop a digital strategy to support universal health coverage (UHC) at a national level and ultimately achieve the health aims of the Sustainable Development Goals (SDGs) [1,2].

Amid the heightened interest, digital health innovations (DHI) have often been rolled out without examining the evidence, resulting in a proliferation of short-lived projects and diverse digital tools [3,4]. Consequently, there is limited understanding or definitive consensus on the impact of DHIs on health systems and health outcomes [5–7]. This concern was highlighted by the WHO Bellagio eHealth Evaluation Group, which stated, “To improve health and reduce health inequalities, rigorous evaluation of eHealth is necessary to generate evidence and promote the appropriate integration and use of technologies” [8].

While recognising the innovative role digital technologies can play in strengthening health systems, there is a need to understand their contribution better and generate evidence to support future investment decisions. This is most important in low- and lower-middle-income countries (LLMIC), where health service delivery is resource-sensitive and where digital health must add demonstrable benefits to justify the allocation of resources that could otherwise be spent on facilities, equipment, staff, medicines, and other essential commodities [6,7].

In October 2023, to articulate what DHIs can be used to overcome health system challenges, WHO produced the *Classification of Digital Interventions, Services and Applications in Health* (CDISAH) [9]. In the CDISAH, DHIs are organised into four categories based on their primary user group: (i) persons (i.e., consumers of healthcare services), (ii) healthcare providers, (iii) healthcare managers and support personnel, and (iv) data service managers.

This protocol has been written in line with the Preferred Reporting Items for Systematic Review and Meta-Analysis Protocols (PRISMA-P) checklist (S1 File) [10].

## Objectives

This review will explore the evidence for the effective use of DHIs by ‘healthcare providers’ (the second group in the CDISAH) in LLMICs to address health system challenges. Our objective is to provide a synthesis of evidence about how DHIs have been used to good effect to support health service delivery in LLMICs.

## Method

Given the plethora of published literature on DHIs, an umbrella review will be conducted. An umbrella review is a “systematic collection and assessments of multiple systematic reviews and meta-analyses done on a specific research topic” [11].

We will follow the umbrella review process developed by Arksey and O’Malley, which involves five steps: (1) identification of research question/s; (2) identification of relevant studies; (3) study screening and selection; (4) charting the data; and (5) collating, summarising, synthesising, and reporting results [12].

The review will be undertaken in accordance with the PRISMA-P guidelines (S1 File) [13].

**Fig 1:**
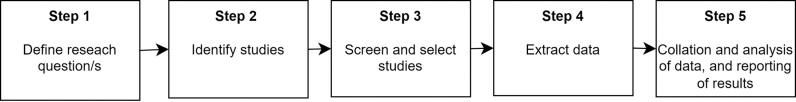
Umbrella review steps.

The review will involve five steps (Fig 1).

### Step 1: Research questions

The review will seek answers to two questions:

- How are healthcare providers in LLMIC using DHIs to address health system challenges (as outlined in the CDISAH)?
- What is the quality of the systematic reviews being analysed?

We use the Problem, Intervention, Comparison, and Outcome (PICO) framework to develop the research questions. Each component is defined below.

- Problem: Inefficient, ineffective, or inequitable delivery of, or access to healthcare
- Intervention: DHIs used to support health systems needs
- Comparison: The status quo or non-digital approaches to health service delivery
- Outcome: Change to access, effectiveness, efficiency, or reach of health services or impact on health equity.

For this review, a healthcare provider will be defined as a member of the health workforce who delivers health services, including doctors, nurses, community health providers, allied health professionals, and health volunteers. DHIs will be defined as digital and mobile technologies used to support health system needs [14]. Low and low-middle-income countries will be those countries identified as such in the World Bank’s ‘Country Classifications by Income Level: 2024–2025’ [15].

### Step 2: Identifying studies

A predefined search strategy encompassing a combination of keywords with Boolean operators in the following syntax will be used. The syntax will include ‘Digital Health’ (and related terms) AND ‘Universal Health Coverage’ (and related terms)] AND ‘LLMICs’ (and related terms).

Four electronic databases—PubMed, Embase, SCOPUS, and Web of Science—will be searched through the Ovid interface. Limiters applied will include ‘humans,’ ‘English,’ ‘published between January 2010 and August 2024 (approximately 15 years), and ‘review articles only.’

**Fig 2:**
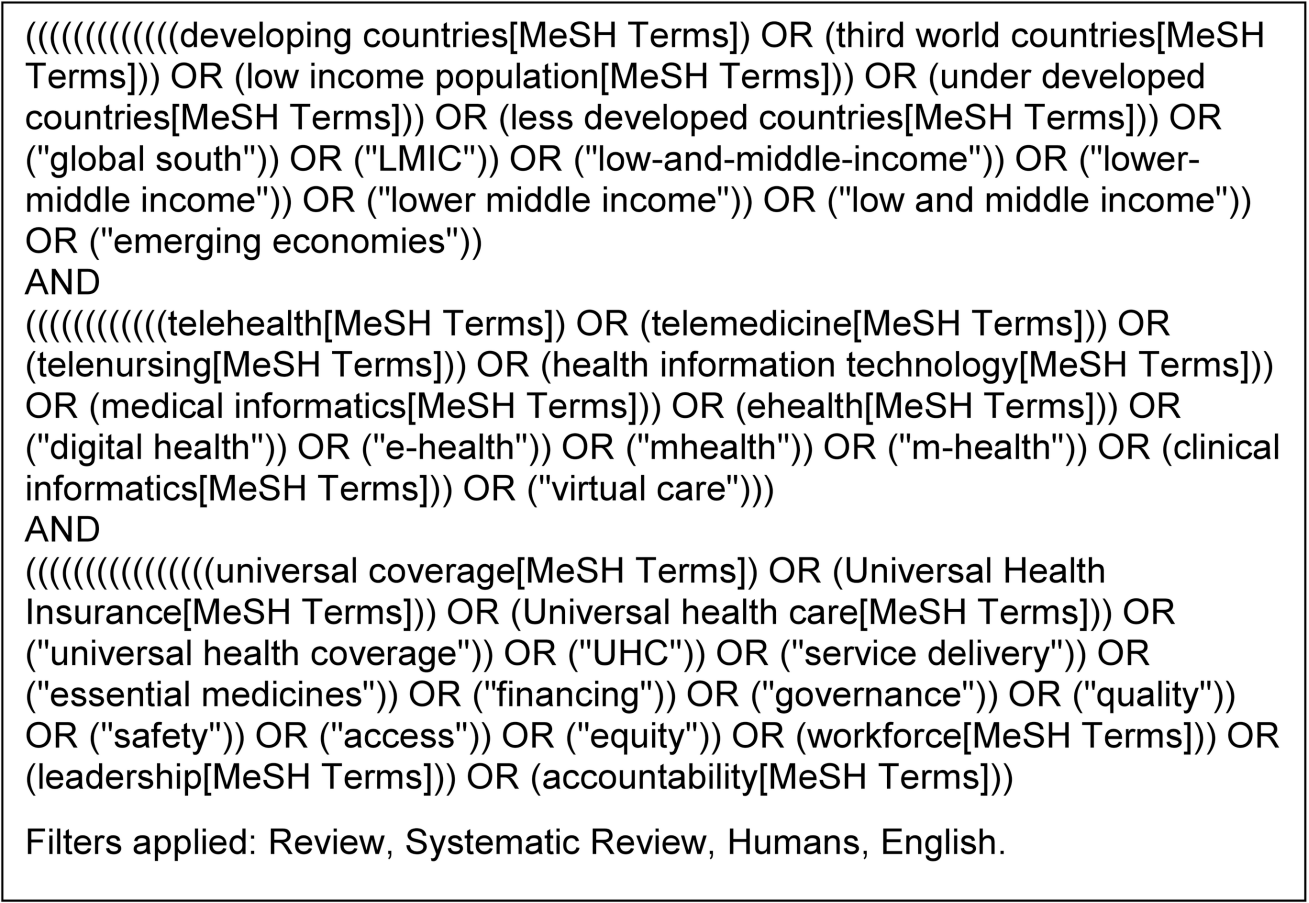
Preliminary literature review search strategy using PubMed taxonomy.

Fig 2 provides a preliminary search strategy developed for PubMed.

### Step 3: Screen and selection studies

#### Inclusion and exclusion criteria.

An article must meet the following criteria to be included in the review. Articles must (i) be review articles; (ii) describe a systematic data identification and extraction method; (iii) relate to a DHI implemented in an LLMIC; (v) be available in English; and (vi) be published between January 2010 and August 2024.

Studies will be excluded if they are not presented as review articles, do not include a description of the article screening and selection method, if the method used was not systematic, or if the DHIs discussed were not applied in LLMIC contexts.

#### Study selection.

A two-stage study selection process will be applied.

First, two authors will independently screen the title and abstract of articles using the abovementioned inclusion and exclusion criteria. Articles with which the reviewers disagreed were automatically be included in the second (full-text screening) stage.

At the full-text screening stage, the two authors will independently assess potentially eligible references for inclusion. A PRISMA flow diagram will document the excluded references and reasons for full-text exclusions.

The ‘Covidence—Better systematic review management’ software (www.covidence.org/) will support all study selection phases.

### Step 4: Extract data

A standardised data extraction form will be developed and piloted on five studies, with adjustments made as necessary. The extraction will cover study characteristics (title, author, year, journal, study type), details of the DHIs discussed (intervention, location, description), and their impact (both positive and negative) on health service delivery and function. Data related to context and exemplar case studies will also be extracted.

Given the anticipated substantial heterogeneity among the included studies, we will not perform a meta-analysis or meta-synthesis. Instead, we will conduct a narrative inductive thematic synthesis of the extracted data, following the methods outlined by Terry, Hayfield, Clarke, and Braun [16]. This approach involves iterative coding and recoding until dominant themes emerge. Some refer to it as a textual narrative synthesis, which is characterised by using a standardised data extraction process, unlike a simple narrative synthesis [17]. This method has been employed in systematic reviews [18,19].

Emergent themes will then be categorised as per the taxonomy for DHIs designed for healthcare providers outlined in the CDISAH. These are (i) identifying and registering persons, (ii) generating a person-centred health record, (iii) delivering healthcare provider decision support, (iv) telemedicine; (v) healthcare provider communication, (vi) referral coordination, (vii) scheduling and activity planning for healthcare providers, (viii) healthcare provider training, (ix) prescription and medication management, (x) laboratory and diagnostic imaging management and (xi) healthcare provider financial transactions [9].

### Step 5: Collating, analysis and reporting of results

We anticipate publishing the results in a peer-reviewed publication and presenting results at relevant conferences and meetings. Should the opportunity arise, we will also engage in dialogue with policymakers to support knowledge interpretation and translation into policy and practice.

### Systematic review quality assessment

To evaluate the methodological quality and risk of bias, authors will assess all studies using the ‘A MeaSurement Tool to Assess systematic Reviews’ (AMSTAR 2) tool [20,21]. AMSTAR 2 used a rating system based on the presence or absence of critical and non-critical weaknesses across 16 ‘quality’ domains. Each domain is evaluated individually to determine the overall quality of a systematic review. Reviews are not assigned an overall numerical score but instead are categorised into four levels of confidence based on the ratings: high confidence (i.e., no or one non-critical weakness); moderate confidence (more than one non-critical weakness); low confidence (one critical flaw with or without non-critical weaknesses; and critically low confidence (more than one crucial flaw with or without non-critical weaknesses) [20].

### Ethics

This protocol will not evaluate individual patient information or affect patient rights and, therefore, does not require ethical approval.

## Discussion

This umbrella review will explore, synthesise, and evaluate the quality of evidence on how healthcare providers in LLMICs utilise DHIs to address service delivery challenges and their effectiveness. To our knowledge, this review will be the first to provide a comprehensive synthesis of evidence specifically focused on DHIs used by healthcare providers in LLMIC and the first to align with WHO’s taxonomy for DHIs, as outlined in WHO’s 2023 *Classification of Digital Interventions, Services and Applications in Health*.

Our review will be founded on an exhaustive search strategy across multiple reference databases, enabling us to identify strengths and gaps in the existing knowledge related to the use of DHIs. This approach will also help pinpoint avenues for future research and opportunities to enhance practices.

## Limitations

We anticipate significant heterogeneity among the studies. Given the nature of the source literature (i.e., systematic reviews), we expect some important nuances to be overlooked and unavailable to us. Additionally, we appreciated that not all relevant research will be captured in the systematic reviews we draw on, and some studies may appear across multiple reviews. We expect that the findings may underemphasise the importance of local context, dynamics, and champions in implementing and succeeding digital health initiatives.

## Supporting information

S1 File PRISMA-P checklist. (DOCX)
